# Wissensstand und Fehlvorstellungen zu Vorsorgedokumenten – Ergebnisse einer Bürgerbefragung

**DOI:** 10.1007/s00103-023-03751-y

**Published:** 2023-08-17

**Authors:** Carolin Fleischmann, Tanja Henking, Silke Neuderth

**Affiliations:** Institut für Angewandte Sozialwissenschaften, Technische Hochschule Würzburg-Schweinfurt, Münzstr. 12, 97070 Würzburg, Deutschland

**Keywords:** Vorsorgedokumente, Patientenverfügung, Vorausplanung, Entscheidungen am Lebensende, Wissen, Advance directives, Living will, Advance care planning, End-of-life decisions, Knowledge

## Abstract

**Einleitung:**

Vorsorgedokumente wie Patientenverfügung oder Vorsorgevollmacht sind bedeutsame Instrumente der Vorausplanung für Situationen, in denen ein Mensch nicht mehr für sich selbst entscheiden kann. Obwohl immer mehr Bürger:innen in Deutschland solche Dokumente erstellen, ist über ihr Wissen zu Zweck, Arten und Anwendung von Vorsorgedokumenten wenig bekannt. Nach über 10 Jahren seit der gesetzlichen Verankerung der Patientenverfügung soll diese Studie das Wissen von Bürger:innen erfassen und Wissenslücken detektieren.

**Methoden:**

In der Stadt und im Landkreis Würzburg wurde 2021 eine Querschnittsbefragung von volljährigen Bürger:innen u. a. zu Besitz, Umgang mit und Wissensstand zu Vorsorgedokumenten durchgeführt. Die Rekrutierung erfolgte über Werbeanzeigen und lokale Netzwerke.

**Ergebnisse:**

Von den 282 Befragten (M_Alter_ = 50 J., zu 2 Drittel weiblich) hatten 43,4 % nach Selbstangabe zumindest ein Vorsorgedokument verfasst. Im Wissenstest wurden im Mittel 22/34 Punkten erreicht. Fragen zur konkreten Anwendung von Vorsorgedokumenten anhand eines Fallbeispiels sowie zu Sterbehilfearten wurden häufig korrekt beantwortet, wohingegen beim Faktenwissen zu den einzelnen Dokumenttypen größere Wissensdefizite bestanden. Objektiv getestetes Wissen und Variablen zum subjektiven Wissensstand korrelieren positiv.

**Diskussion:**

Die relativ hohe Quote an erstellten Dokumenten in dieser Stichprobe ist Ausdruck ihrer raschen Verbreitung in den letzten Jahren. Das Wissensniveau ist als niedrig einzuschätzen und zeigt Fehlvorstellungen zu Rechten und Pflichten der verfassenden Person sowie der beteiligten Akteure. Das gemessene Wissen der Bürger:innen steht in Diskrepanz zum häufig geäußerten Wunsch, durch informiertes Erstellen von Vorsorgedokumenten ihre Selbstbestimmung zu wahren.

**Zusatzmaterial online:**

Zusätzliche Informationen sind in der Online-Version dieses Artikels (10.1007/s00103-023-03751-y) enthalten.

## Abstract (en)

**Background:**

Advance directives (ADs) such as living wills or healthcare powers of attorney are important tools to anticipate medical treatment decisions when decision-making capacity is lost in the future. Although a rising number of citizens in Germany are creating such documents, little is known about their knowledge of the purpose, types, and use of ADs. After more than 10 years since legislation on ADs came into force, this study intends to measure the objective knowledge of citizens and detect deficits in knowledge.

**Methods:**

We conducted a cross-sectional quantitative survey of citizens aged 18+ in the city and county of Wuerzburg. The questionnaire included, among other things, possession, experience, and knowledge of ADs. Sampling was conducted via advertising and local networking.

**Results:**

Of the 282 participants who took part in the survey (M_age_ = 50 years, 2/3 female), 43.4% reported having created a minimum of one document. In the knowledge test, an average of 22/34 points was achieved. While questions about the specific application of ADs based on a case study were often answered correctly, we found deficits about the single document types. The results in the knowledge test and the variables on the subjective level of knowledge correlate positively.

**Discussion:**

The relatively high rate of ADs in this sample indicates their rapid dissemination during the past few years in Germany. Overall, the level of knowledge ADs appears to be low, revealing misconceptions about the creator’s and involved people’s rights and obligations. The measured knowledge level contradicts with the frequently expressed desire of citizens to preserve their autonomy by creating ADs for themselves.

## Einleitung

Vorsorgedokumente in Form von Patientenverfügung, Vorsorgevollmacht und Betreuungsverfügung spielen eine wichtige Rolle zur Wahrung der Patientenautonomie in Lebens- und Krankheitssituationen, in denen die verfügende Person durch Unfall oder Krankheit dauerhaft oder temporär einwilligungsunfähig ist und keine Entscheidungen treffen kann. Ihre rechtliche Verbindlichkeit ergibt sich aus § 1827 BGB (Bürgerliches Gesetzbuch). Vorsorgedokumente werden sowohl vom medizinischen Personal [1, 2] als auch in der Allgemeinbevölkerung weitgehend akzeptiert und als hilfreich angesehen [3, 4]. Alle 3 Dokumente können als Bündel an Möglichkeiten einer Vorausplanung verstanden werden. Denn auch bei Anwendung einer Vorsorgevollmacht werden medizinische Behandlungsentscheidungen nach dem Patientenwillen getroffen, auch wenn diese nicht in Form einer Patientenverfügung verschriftlicht sind. Die Anzahl an Behandlungsentscheidungen am Lebensende hat in den letzten Jahren im intensivmedizinischen Bereich zugenommen. Solche Entscheidungen betreffen die Frage nach der Fortführung oder Beendigung lebenserhaltender Maßnahmen, aber auch deren Nichtaufnahme oder Begrenzung [5]. Der Einfluss von Patientenverfügungen auf ärztliche Behandlungsentscheidungen, zum Beispiel in Notfallsituationen, bei Intensivbehandlungen oder am Lebensende, brachte bisher allerdings keine einheitlichen Ergebnisse und wird kontrovers diskutiert [6–8].

Der Bekanntheitsgrad und das Vorhandensein von Vorsorgedokumenten sind in den letzten Jahren in Deutschland kontinuierlich angestiegen, wobei sich die Prävalenzen in den untersuchten Stichproben unterscheiden. Eine repräsentative Umfrage des Allensbach-Instituts ergab bereits im Jahr 2014, dass nahezu alle Bürger:innen von einer Patientenverfügung gehört hatten [9]. Die Prävalenzen verfasster Patientenverfügungen werden in aktuellen Studien zwischen 20,4 % und 35,3 % unter Bewohnenden in stationären Pflegeeinrichtungen [10–12], 54 % unter Senior:innen einer Großstadt [13] und 43 % unter Erwachsenen ab 18 Jahren angegeben [14]. Auch das Zentrale Vorsorgeregister verzeichnet jährlich steigende Registrierungen von Vorsorgevollmachten, die in etwa 75 % mit einer Patientenverfügung verbunden sind [15, 16].

Auf der einen Seite entscheiden sich also immer mehr Menschen für eine schriftliche Vorsorge in Form einer Vorausverfügung, auf der anderen Seite begegnen motivierten Bürger:innen bei der Erstellung eines solchen Dokuments zahlreiche Hürden, allen voran die medizinische, ethische und rechtliche Komplexität. Beispielsweise drehen sich ethische Fragen um Chancen und Risiken einer antizipierten Willensbestimmung, die rechtlichen Fragen um die Anforderungen an (nicht) eindeutige Formulierungen in Patientenverfügungen [17]. Über den Informationsstand zu Vorsorgedokumenten in der deutschen Allgemeinbevölkerung ist wenig bekannt. Bereits vor Inkrafttreten des „Patientenverfügungsgesetzes“ bestand großes Interesse an den Möglichkeiten einer Vorausverfügung [18]. Die gesetzliche Verankerung der Patientenverfügung im Jahr 2009 bedarf jedoch einer neuen Bewertung der wissenschaftlichen Aufarbeitung. Elmeadawy et al. hatten im Jahr 2017 Patient:innen einer allgemeininternistischen Station befragt und als Gründe für das Nichtvorhandensein von Vorsorgedokumenten unter anderem eine geringe Informiertheit zum Thema und zum Erstellungsprozess gefunden. Nur 21 % hatten von den seit 2009 bestehenden gesetzlichen Rahmenbedingungen zu Vorsorgedokumenten gehört [19]. Das Internet hält zahlreiche private, gemeinnützige oder staatliche Informations- und Unterstützungsangebote bereit. Häufig werden in einem Frage-Antwort-Stil Fehlannahmen zu Vorsorgedokumenten berichtigt, wie beispielsweise die Notwendigkeit einer notariellen Beglaubigung [19–21]. Die gleichen Herausforderungen finden sich international im Hinblick auf die zu berücksichtigenden unterschiedlichen rechtlichen Rahmenbedingungen [22, 23].

Diese Studie hat zum Ziel, erstmals in Deutschland den subjektiven und objektiven Wissensstand von Bürger:innen zu den in Deutschland üblichen Vorsorgedokumenten Patientenverfügung, Vorsorgevollmacht und Betreuungsverfügung zu erheben. Angesichts der jährlich steigenden Anzahl und der seit mehr als 10 Jahren bestehenden gesetzlichen Regelung der Patientenverfügung ist diese Analyse notwendig, um das Wissensniveau einschätzen zu können, Fehlvorstellungen zu detektieren sowie Informationsangebote bedarfsgerecht zu gestalten.

## Methoden

### Studiendesign

Von Juli bis Dezember 2021 fand eine Querschnittsbefragung von volljährigen Bürger:innen in der Stadt und im Landkreis Würzburg statt. Die Befragung erfolgte online über die Webseite Unipark sowie auf Anforderung als Paper-Pencil-Version. Bei beiden Versionen war kein Rückschluss auf personenbezogene Daten möglich. Ein Ethikvotum war nach Anfrage bei der Ethikkommission der Universität Würzburg aufgrund der Anonymität der Umfrage nicht erforderlich (AZ: 20201117 01). Die Teilnehmenden wurden vor Umfragebeginn über Ziele und relevante Eckdaten der Studie sowie die Datenverarbeitung informiert.

### Rekrutierungsstrategie

Die Rekrutierung umfasste umfangreiche Werbeaktionen über unterschiedliche Medien und Netzwerke, um Zugang zu allen Altersgruppen und Bildungsschichten zu gewährleisten. Dafür wurden Teilnahmeaufrufe über Social Media gestreut sowie als klassische Printwerbeanzeigen und -beilagen in lokalen Zeitungen und Magazinen gedruckt. Für den Zugang zur älteren, digital naiven Bevölkerung wurde die Umfrage zudem beispielsweise über die Seniorenbeiräte des Kommunalunternehmens gestreut.

### Erhebungsinstrument

Validierte deutschsprachige Instrumente zur Wissenserfassung in Bezug auf Vorsorgedokumente liegen nicht vor. Innerhalb eines interdisziplinären Teams mit medizinischer, juristischer und psychologischer Expertise wurde ein Fragebogen entwickelt, basierend auf einer Recherche zu häufig anzutreffenden Fehlvorstellungen über Vorsorgedokumente. Der 5‑teilige Fragebogen umfasst weitgehend geschlossene Fragen zu Soziodemographie (Teil 1), Kenntnis und Besitz von Vorsorgedokumenten (Teil 2) und subjektivem Informationsstand (Teil 5). Darüber hinaus werden die Einschätzung der eigenen Kompetenzen und praktischen Fähigkeiten zu Vorsorgedokumenten sowie Informationsbedarfe erfragt (Teil 3). Das Kernelement bildet ein objektiver Wissenstest mit 34 One-Choice-Fragen mit den Antwortoptionen: „richtig“, „falsch“ und „weiß nicht“ (Original-Wissenstest als Onlinematerial, Teil 4). 22 Fragen zielen auf substanzielle Kenntnisse zu Patientenverfügung, Vorsorgevollmacht und Betreuungsverfügung ab sowie auf Fehlvorstellungen zu deren Zweck, Anwendung, Reichweite und auf notwendige Formalien. Mittels eines adaptierten Fallbeispiels [24] wurde mit 9 Fragen das Verständnis für die realen Auswirkungen der Anwendung von Vorsorgedokumenten überprüft. Anhand von 3 weiteren fiktiven Fallbeschreibungen wurde die rechtliche Zulässigkeit verschiedener Sterbehilfearten abgefragt. Im Anschluss wurde die subjektive Erfolgseinschätzung bei der Beantwortung der Fragen erfasst. Nach Durchführung eines Pretests mit 11 Personen wurden einzelne Fragen angepasst, um unterschiedliche Schwierigkeitsgrade abzudecken.

Die Datenauswertung erfolgte deskriptiv mit SPSS 28. Die den statistischen Tests zugrunde liegende Irrtumswahrscheinlichkeit liegt bei α < 0,05 bei zweiseitiger Testung.

## Ergebnisse

Insgesamt beteiligten sich 286 Bürger:innen, wovon 271 auf die Online-Version (Beendigungsquote von 10 % bei 2673 Aufrufen der Online-Version) und 15 auf die Papierversion entfielen. 4 Fälle wurden aufgrund vollständig fehlender Angaben gelöscht. Demnach standen 282 Fälle zur Auswertung zur Verfügung.

### Stichprobe

Es nahmen fast doppelt so viele Frauen (*n* = 179) wie Männer (*n* = 98) an der Befragung teil (Tab. 1). Das durchschnittliche Alter liegt bei 50 Jahren bei einer Altersspanne zwischen 18 und 87 Jahren (Md = 53 J.; SD = 16 J.). 28,7 % Personen haben einen akademischen Bildungsabschluss. Der Großteil der Befragten (*n* = 220; 78,6 %) hat seinen Wohnsitz in der Stadt und im Landkreis Würzburg, weitere 47 (16,5 %) in daran angrenzenden Landkreisen oder im Regierungsbezirk Unterfranken. Die vorherrschende Wohnform ist der 2‑Personen-Haushalt in Partnerschaft. Bis auf 10 Personen leben alle Befragten seit ihrer Geburt in Deutschland.

**Table 1:** 

Variable (gültige Werte: *n*)	Häufigkeit (*n*)	Prozent (%)
*Wohnort (n* *=* *273)*		
Stadt oder Landkreis Würzburg	220	78
An Würzburg angrenzende Landkreise	38	13,5
Landkreis in Unterfranken	9	3,2
Landkreis außerhalb Unterfrankens	7	2,5
*Geschlecht (n* *=* *277)*		
Männlich	98	35,4
Weiblich	179	64,6
*Alter (n* *=* *282)*		
Unter 20 Jahren	1	0,4
20–39 Jahre	75	26,6
40–59 Jahre	116	41,1
60–79 Jahre	81	28,7
80 Jahre und mehr	9	3,2
*Wohnform (n* *=* *282)*		
Alleinlebend	44	15,6
Mit Partner/Partnerin zusammenlebend	132	46,8
In Wohngemeinschaft lebend	19	6,7
Mit Familie in 2 Generationen zusammenlebend	82	29,1
Mit Familie in mind. 3 Generationen zusammenlebend	5	1,8
*Familienstand (n* *=* *281)*		
Ledig	69	24,6
Verheiratet/eingetragene Lebenspartnerschaft	178	63,3
Geschieden	19	6,8
Verwitwet	15	5,3
*Höchster Bildungsabschluss (n* *=* *282)*		
Hochschulabschluss	57	20,2
Fachhochschulabschluss/Hochschule für angewandte Wissenschaften	24	8,5
Fachhochschulreife/Abitur und berufliche Ausbildung	48	17,0
Fachhochschulreife/Abitur ohne berufliche Ausbildung	13	4,6
Mittlere Reife und berufliche Ausbildung	94	33,3
Mittlere Reife ohne berufliche Ausbildung	3	1,1
Hauptschulabschluss und berufliche Ausbildung	38	13,5
Hauptschulabschluss ohne berufliche Ausbildung	3	1,1
Kein Abschluss	2	0,7

### Angaben zu Vorsorgedokumenten

#### Kenntnis und Besitz

Nahezu alle Befragten haben schon einmal von einer Patientenverfügung gehört, 84 % von der Vorsorgevollmacht und nur 61 % von der Betreuungsverfügung (*n* = 172; Tab. 2). 43,4 % (*n* = 122) besitzen nach eigener Auskunft selbst eines der 3 Dokumente, und zwar zu 88,1 % (*n* = 104) eine Patientenverfügung und an zweiter Stelle zu 83,1 % (*n* = 98) eine Vorsorgevollmacht. Der Altersdurchschnitt der Befragten, die ein Vorsorgedokument besitzen, liegt 11 Jahre über dem der Personen ohne vorhandene Vorsorgedokumente (56,7 vs. 45,6 Jahre; *p* > 0,001). Die Besitzquote steigt mit dem Alter kontinuierlich an (r_pb_ = 0,341; *p* < 0,001). Als häufigster Grund für das Nichtvorhandensein von Patientenverfügung oder Vorsorgevollmacht wird das Aufschieben des Themas genannt. Weitere häufig genannte Gründe sind Zeitmangel und mangelnde Beschäftigung mit dem Thema. Bei der Patientenverfügung steht bei 40 % (*n* = 68 von 172 ohne Patientenverfügung) außerdem die Unsicherheit bei der Erstellung im Vordergrund.

**Table 2:** 

Variable (gültige Werte *n*)	Häufigkeit (*n*)	Relativer Anteil (%)
**1. Kenntnis, Besitz und Erfahrung mit Vorsorgedokumenten**		
*Kenntnis über Vorsorgedokumente (n* *=* *282)*		
Patientenverfügung	281	99,9
Vorsorgevollmacht	237	84,0
Betreuungsverfügung	172	61,0
*Besitz von Vorsorgedokumenten (n* *=* *281)*		
Ja	122	43,4
Nein	159	56,6
*Wenn ja, welche/s Dokument/e besitzen Sie? (n* *=* *118)*		
Patientenverfügung	104	88,1
Vorsorgevollmacht (auch Generalvollmacht)	98	83,1
Betreuungsverfügung	47	39,8
*Waren Sie schon einmal in einer Situation, in der Sie für sich oder eine nahestehende Person ein Vorsorgedokument benötigt haben/hätten? (n* *=* *282)*		
Ja	114	40,4
Nein	168	59,6
*Falls Sie keine Patientenverfügung haben: Warum nicht? (von mind. 5* *% gewählte Antworten, n* *=* *172)*		
Ich habe bislang nicht darüber nachgedacht	33	19,2
Bislang keine Zeit dafür gefunden	61	35,5
Bin noch zu jung dafür/das ist eher etwas für ältere Menschen	17	9,9
Habe keine (ernsthaften) Erkrankungen/bin gesund	28	16,3
Ich habe Angst, dann nicht richtig behandelt zu werden	11	6,4
Ich weiß nicht/bin unsicher, wie man das macht	68	39,5
Ich schiebe das Thema vor mir her	116	67,4
Das Thema belastet mich/ist mir unbehaglich	17	9,9
*Falls Sie keine Vorsorgevollmacht haben: Warum nicht? (von mind. 5* *% gewählte Antworten, n* *=* *180)*		
Ich habe bislang nicht darüber nachgedacht	43	24,4
Bislang keine Zeit dafür gefunden	53	30,1
Ich wusste nicht, dass es sowas gibt	13	7,4
Ich will nicht die Kontrolle über meine Angelegenheiten abgeben	10	5,7
Bin noch zu jung dafür/das ist eher etwas für ältere Menschen	14	8,0
Habe keine (ernsthaften) Erkrankungen/bin gesund	18	10,2
Ich schiebe das Thema vor mir her	106	60,2
Das Thema belastet mich/ist mir unbehaglich	16	9,1
*Wie schätzen Sie die Bedeutung von Vorsorgedokumenten ein? (n* *=* *281)*		
Niedrig	3	1,1
Eher niedrig	7	2,5
Eher hoch	124	44,1
Hoch	147	52,3
**2. Beratung und Informationssuche**		
*Bereits fachliche Beratung zu Vorsorgedokumenten wahrgenommen? (n* *=* *282)*		
Ja	76	26,6
Nein	204	72,3
*Bei wem Beratung wahrgenommen? (n* *=* *76)*		
Rechtsanwält:innen/Notar:innen	33	43,4
Ärzt:innen	22	28,9
Gemeinnützige/kommunale Beratungsstellen	16	21,1
Unabhängige Patientenberatung Deutschland (UPD)	2	2,6
Verbraucherzentrale	6	7,9
Andere (Vorträge, Familie, andere Berater)	15	19,7
*Bei wem würden Sie sich beraten lassen? (n* *=* *279)*		
Ärzt:innen	145	52,0
Gemeinnützige/kommunale Beratungsstellen	123	44,1
Unabhängige Patientenberatung Deutschland (UPD)	69	24,7
Verbraucherzentrale	42	15,1
Rechtsanwält:innen/Notar:innen	121	43,4
Andere (Internet, Bank, Hospiz, Betreuungsstelle, Freunde)	16	5,7
**3. Informationsbedarf**		
*Haben Sie generell den Wunsch, mehr über Vorsorgedokumente zu erfahren? (n* *=* *281)*		
Überhaupt nicht	4	1,4
Eher nicht	19	6,8
Eher schon	127	45,2
Auf jeden Fall	131	46,6
*Warum würden Sie ein Vorsorgedokument erstellen wollen? (von mind. 5* *% gewählte Antworten, n* *=* *282)*		
Ich möchte selbst bestimmen, was mit mir geschieht	246	87,2
Ich will meine Angehörigen entlasten	226	80,1
Mein Sterben soll nicht unnötig/künstlich verlängert werden	212	75,2
Da ich im Bekannten‑/Verwandtenkreis Verläufe miterlebt habe, die ich so nicht erleben will	62	22,0
Das ist einfach wichtig heutzutage	138	48,9
In meinem Alter sollte man das haben	84	29,8
*Von wem sollte der Impuls zur Erstellung ausgehen? (n* *=* *281)*		
Von mir selbst	271	96,4
Aus der Familie	79	28,1
Hausärzt:innen	76	27,0
Krankenkassen/Pflegeberatung	62	22,1
Sonstige	6	2,1

#### Informationsstand und Erfahrung

Der subjektive Informationsstand als Antwort auf die Frage: „Wie gut fühlen Sie sich zu Vorsorgedokumenten informiert?“, liegt auf einer Skala von 1–10 (1 = gar nicht gut, 10 = sehr gut) im Mittel bei 5 (Md = 5; SD = 2,8). 114 Teilnehmende (40,4 %) geben an bereits in einer Situation gewesen zu sein, in der ein Vorsorgedokument benötigt wurde. Die Bedeutung der Vorsorgedokumentation wurde auf einer 4‑stufigen Likert-Skala (niedrig–hoch) von 96,4 % (*n* = 271) der Befragten als hoch oder eher hoch eingeschätzt (Tab. 2).

#### Beratung und Beschaffung von Informationen

Von den 76 Personen, die bereits einmal eine fachliche Beratung zu Vorsorgedokumenten wahrgenommen hatten (entspricht 27 %), geben 33 an, diese bei Rechtsanwält:innen oder Notar:innen in Anspruch genommen zu haben (Tab. 2). An zweiter und dritter Stelle werden Ärzt:innen und Beratungsstellen genannt. Alle 282 Bürger:innen wurden gefragt, bei wem sie sich hypothetisch beraten lassen würden. Sie geben zu 52 % Ärzt:innen, zu 44,1 % Beratungsstellen und zu 43,4 Anwält:innen/Notar:innen an (*n* = 279; 3 Angaben fehlend).

### Einschätzung der eigenen Kompetenzen und praktischen Fähigkeiten

**Figure 1:**
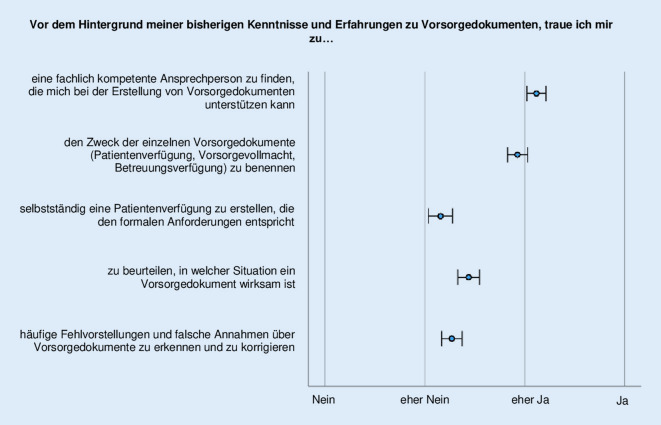

Auf einer 4‑stufigen Skala (1 = Nein, 2 = eher Nein, 3 = eher Ja, 4 = Ja) sollten die Teilnehmenden ihre subjektiven Kompetenzen und Fähigkeiten im Kontext von Vorsorgedokumenten einschätzen (Abb. 1). Am wenigsten trauen sie sich demnach zu, selbst eine Patientenverfügung zu erstellen (M = 2,2; SD = 1) sowie Fehlvorstellungen zu erkennen und zu korrigieren (M = 2,3; SD = 0,9). Das relativ betrachtet höchste Zutrauen haben sie beim Finden einer kompetenten Ansprechperson (M = 3,1; SD = 0,8).

### Wissen zu Vorsorgedokumenten und Sterbehilfe

Die durchschnittlich erreichte Punktzahl im Wissenstest liegt bei 22 von 34 möglichen Punkten (*n* = 257; SD = 4,7; Md = 23; Min. = 0; Max. = 32), was 65 % der maximal erreichbaren Punktzahl entspricht. Die höchsten Fehlerquoten sind bei den detaillierteren Fragen zu den einzelnen Dokumenttypen zu finden, mit entsprechend niedrigeren durchschnittlichen Quoten korrekter Antworten pro Abschnitt (8 Fragen zur Patientenverfügung: 46 %; 4 Fragen zur Vorsorgevollmacht: 56 %; 3 Fragen zur Betreuungsverfügung: 47 %).

Aus dem Antwortverhalten können Themenbereiche identifiziert werden, die durch Häufungen von falschen Antworten auf Fehlannahmen hindeuten bzw. bei häufigem Wählen der Option „weiß nicht“ auf Nichtwissen. Die Quoten korrekter Antworten unter den 34 Fragen reichen von 6,8–96,8 %, die Quoten falscher Antworten von 0,4–86,5 %; die nicht gewusster Antworten von 2,1–38,7 %. Besonders hohe Quoten mit > 60 % *nicht korrekter* Antworten weisen Fragen über folgende Inhalte auf (Tab. 3):

- ärztliche Möglichkeiten der Therapiebegrenzung bei fehlender Patientenverfügung (bei 93,3 % nicht korrekt),
- Möglichkeiten von Menschen mit einer Demenzerkrankung, Vorsorgedokumente zu erstellen (bei 74,5 % nicht korrekt),
- Zulässigkeit des Abschaltens des Beatmungsgeräts bei fehlender Indikation (bei 64,9 % nicht korrekt),
- Option der Ablehnung wie auch Zustimmung von/zu Behandlungsmaßnahmen in einer Patientenverfügung (bei 61,7 % nicht korrekt).

**Table 3:** 

Frage^a^	Frageninhalt	Lösung	Antwort fehlend *n* (%)	Korrekt beantwortet *n* (%)	Nicht korrekt beantwortet *n* (%)		
					Falsche Antwort	Nicht gewusst	Gesamt
33	Erstellung eines VD bei Demenz	Falsch	–	72 (25,5)	101 (35,8)	109 (38,7)	210 (74,5)
35	Lebenserhaltung bei Nichtvorliegen eines VD	Falsch	3 (1,1)	19 (6,8)	243 (86,5)	17 (6,8)	260 (93,3)
36	Bestätigung durch Zeugen bei PV	Falsch	1 (0,4)	136 (48,4)	78 (27,8)	67 (23,8)	145 (51,6)
38	PV steht über aktuellem Willen	Falsch	–	142 (50,4)	79 (28,0)	61 (21,6)	140 (49,6)
39	Fehlende Gesetze zur PV	Falsch	–	98 (34,8)	50 (17,7)	134 (34,8)	184 (52,5)
41	PV-Gültigkeit ohne Aktualisierung	Richtig	1 (0,4)	148 (52,5)	45 (16,0)	88 (31,2)	133 (47,2)
42	Nur Ablehnung von Maßnahmen in PV	Falsch	–	108 (38,3)	100 (35,5)	74 (26,2)	174 (61,7)
43	Keine Betreuerbestellung, wenn VV vorliegt	Richtig	1 (0,4)	156 (55,3)	67 (23,8)	58 (20,6)	125 (44,4)
45	Entscheidungsgewalt Bevollmächtigte über lebenserhaltende Maßnahmen	Richtig	2 (0,7)	147 (52,1)	68 (24,1)	65 (23,0)	133 (47,1)
46	Freiheitsentziehende Maßnahmen in VV	Richtig	–	117 (41,5)	75 (26,6)	90 (31,9)	165 (58,5)
47	Betreuung = Entmündigung	Falsch	1 (0,4)	87 (30,9)	87 (30,9)	72 (25,5)	159 (56,4)
48	Freie Wahl des Betreuers in BV	Richtig	1 (0,4)	156 (55,3)	67 (23,8)	58 (20,6)	125 (44,4)
49	Betreuungsgericht bei Uneinigkeit	Richtig	–	114 (40,4)	59 (20,9)	109 (38,7)	168 (59,6)
59	Fallbeispiel zur Sterbehilfe durch Behandlungsabbruch	Zulässig	2 (0,7)	97 (34,4)	131 (46,5)	52 (18,4)	183 (64,9)

*VD* Vorsorgedokument, *PV* Patientenverfügung, *VV* Vorsorgevollmacht, *BV* Betreuungsverfügung

^a^Fragennummer bezogen auf gesamten Fragebogen

Fragen mit den höchsten Quoten an *nicht gewussten* Antworten („weiß nicht“) drehen sich wiederum um die Möglichkeiten von Menschen mit einer Demenzerkrankung, Vorsorgedokumente zu erstellen (von 38,7 % nicht gewusst), aber auch um die Rolle des Betreuungsgerichts bei Uneinigkeit zwischen Behandelnden und Betreuern (ebenfalls 38,7 %), um die gesetzliche Grundlage von Patientenverfügungen (34,8 %) und um die Gültigkeit einer Patientenverfügung trotz fehlender Aktualisierung (31,2 %).

Fragen mit hohen Quoten *falscher* Antworten betreffen wiederum die ärztlichen Möglichkeiten der Therapiebegrenzung bei fehlender Patientenverfügung (86,5 %) und die Zulässigkeit des Abschaltens des Beatmungsgeräts bei fehlender Indikation (46,5 %), aber auch die korrekte Zuordnung der vorgegebenen Definitionen von Vorsorgevollmacht und Betreuungsverfügung, welche jeweils von rund einem Drittel der Befragten verwechselt werden (VV: 32,7 % falsch; BV: 30 % falsch; nicht in Tab. 3 dargestellt), die Verwendung des Begriffs „Entmündigung“ bei Betreuung (30,9 %) und die Rangfolge von aktuellem Willen und in einer Patientenverfügung verfügtem Willen (28,0 %).

Die Wissensfragen zum Fallbeispiel wiesen durchgehend hohe Quoten korrekter Antworten auf. Am konkreten Beispiel einer 81-jährigen Frau mit mehreren Schlaganfällen und der anstehenden Entscheidung über das Legen einer Nahrungssonde durch die Bauchdecke (perkutane endoskopische Gastrostomie – PEG) konnten die Teilnehmenden im Mittel zu 86 % richtig einschätzen, welche Konsequenz das Vorhandensein von Vorsorgedokumenten auf die Entscheidung hätte, unter welchen Voraussetzungen eine Patientenverfügung wirksam wird und welche Sterbehilfearten in Deutschland zulässig sind.

Der Einfluss verschiedener Variablen auf das Ergebnis im Wissenstest wurde explorativ untersucht. Dabei wurden die Mittelwerte verglichen und die Unterschiede mittels t‑Tests und Effektstärkenberechnungen verifiziert. Demnach haben die 3 Variablen, die am ehesten für eine größere Erfahrung mit Vorsorgedokumenten sprechen, einen signifikanten Einfluss auf das Wissen: der Besitz von Vorsorgedokumenten (23,3 Punkte vs. 21,1 Punkte bei Bürger:innen ohne Vorsorgedokumente; *p* < 0,001; Cohens d = 0,48), die Erfahrung mit der Anwendung von Vorsorgedokumenten (22,9 Punkte vs. 21,4 Punkte bei Bürger:innen ohne Anwendungserfahrung; *p* = 0,011; Cohens d = 0,33) und die Inanspruchnahme einer fachlichen Beratung in der Vergangenheit (23,5 Punkte vs. 21,6 Punkte bei Bürger:innen ohne Beratung; *p* = 0,005; Cohens d = 0,41).

Nach Abschluss des Wissenstests sollten die Befragten in einer Selbsteinschätzung angeben, welchen Anteil der Fragen sie glaubten richtig beantwortet zu haben und welche Anteile davon sie geraten oder durch Vorwissen richtig beantwortet hatten. Im Durchschnitt hielten die Befragten 58 % ihrer Antworten für richtig (*n* = 279; SD = 21 %, Md = 60 %), davon 31 % für geraten und 68 % für durch Vorwissen richtig beantwortet (nur Auswertung korrekter oder nachvollziehbarer Prozentangaben bei *n* = 231). 10 % der Befragten erschien die Beantwortung der Fragen „schwer“, 45,4 % „eher schwer“, 40,8 % „eher leicht“ und 3,6 % „leicht“ (M = 2,38; SD = 0,7). Die Parameter zur Selbsteinschätzung des Wissensstands zu Vorsorgedokumenten zeigen alle signifikant positive Korrelationen mit dem Ergebnis im Wissenstest (Tab. 4).

**Table 4:** 

					95 %-KI	
Variable	Wertebereich	*n*	r	*p*	Unten	Oben
Subj. Schwierigkeitsgrad der Fragen	1–4 (schwer–leicht)	256	0,318	< 0,001*	0,242	0,389
Subjektiver Informationsstand	1–10 (gar nicht gut–sehr gut [informiert])	257	0,389	< 0,001*	0,28	0,488
Summenwert der Kompetenz-Skala	5–20	257	0,422	< 0,001*	0,316	0,518
Selbstgeschätzte Erfolgsquote	0–100 (%)	257	0,413	< 0,001*	0,306	0,510

*KI* Konfidenzintervall

### Informationsbedarf

Informationsbedarf besteht bei 91,8 % (*n* = 258) der Befragten „eher schon“ oder „auf jeden Fall“, und zwar von 75 % (*n* = 209) zu allen 3 Dokumenttypen (Tab. 2). Medizinische Inhalte werden dabei von 57,1 % (*n* = 149/261) und rechtliche Inhalte von 49,2 % (*n* = 129) als „sehr wichtig“ angesehen. Bei der Frage nach den häufigsten Gründen für eine Erstellung von Vorsorgedokumenten sind 3 Argumente führend: der Wunsch nach Selbstbestimmung, die Entlastung der Angehörigen und die Ablehnung unnötiger/künstlicher Lebensverlängerung. Der Impuls zur Erstellung eines Vorsorgedokuments soll aus Sicht der meisten Befragten (96,4 %; *n* = 271) von ihnen selbst bzw. von der Familie (28,1 %; *n* = 79) ausgehen, weniger von Hausärzten oder Kranken- bzw. Pflegekassen.

## Diskussion

### Bekanntheit und Besitz von Vorsorgedokumenten

Der Bekanntheitsgrad von nahezu 100 % in Bezug auf Patientenverfügungen korrespondiert mit der Literatur, in der eine Zunahme der Bekanntheit von Vorsorgedokumenten über die letzten 20 Jahre beschrieben wird [2, 9]. Dass gemäß den Selbstangaben 43,4 % der Befragten im Besitz von Vorsorgedokumenten sind, unterstreicht die zunehmende Prävalenz in der Bevölkerung und fügt sich in die Befunde neuerer Erhebungen ein [13, 14]. Die Prävalenz steigt auch in der vorliegenden Stichprobe mit zunehmendem Alter an [3, 10, 12, 25, 26]. Unsere Befunde bekräftigen die starke Zunahme des Bekanntheitsgrads und der Prävalenz von Vorsorgedokumenten in der Allgemeinbevölkerung.

### Bewertung des Wissens zu Vorsorgedokumenten

Im Durchschnitt wurden 65 % der Fragen im Wissenstest korrekt beantwortet, wobei sich eine große Varianz zwischen einzelnen Wissensinhalten zeigt. Fraglich ist, ob dieses Wissensniveau für die von Bürger:innen beabsichtigten Handlungen, z. B. die Erstellung von Vorsorgedokumenten, ausreichend ist. Der selbsteingeschätzte Informationsgrad, das eher geringe Zutrauen in die selbstständige Erstellung von Vorsorgedokumenten sowie die erlebte Schwierigkeit bei der Beantwortung der Wissensfragen lassen daran zweifeln. Der Informationsbedarf ist selbst bei Personen mit bereits erstellten Vorsorgedokumenten überwältigend hoch. Für eine realistische Selbsteinschätzung der Kenntnisse sprechen die positiven Korrelationen zwischen dem Ergebnis im Wissenstest und den Variablen zur subjektiven Kompetenzeinschätzung. Vergleichbar zu den Befunden anderer Studien war unzureichendes Wissen ein häufiger Grund für das Nichtverfassen einer Patientenverfügung [19, 27]. Der Wunsch, durch Vorausplanung das Recht auf Selbstbestimmung zu wahren [2, 3], kann somit durch mangelndes Wissen wesentlich beeinträchtigt sein.

An die Patientenverfügung als Instrument der antizipierten Willenserklärung sollte bei der Aufklärung/Informationsvermittlung ein ebenso hoher Maßstab wie an jede Art der informierten Einwilligung gesetzt werden. Gleiches gilt schließlich auch für die Vorsorgevollmacht, wenn sie zur Umsetzung des (mutmaßlichen) Willens möglichst in Deckung zum wahren Willen der Person stehen soll. Die Falschantworten auf Fragen zu substanziellem Wissen über die 3 wichtigsten Dokumenttypen offenbarten ein grundlegendes Unwissen über diesbezügliche notwendige Formalien und gesetzliche Bestimmungen sowie über die konkreten Inhalte und damit bestimmbaren Aspekte und die Rechte der im Entscheidungsprozess beteiligten Personen wie rechtliche Vertreter:innen oder behandelnde Ärzt:innen. Verschiedene Fehlvorstellungen sind verbreitet, wie etwa, dass ohne Patientenverfügung die Einstellung einer lebenserhaltenden Therapie nicht möglich wäre, dass es kein Gesetz zur Verbindlichkeit der Patientenverfügung gäbe, dass innerhalb einer Patientenverfügung nur Maßnahmen abgelehnt werden könnten, dass eine Betreuung einer „Entmündigung“ entspräche oder dass in einer Patientenverfügung verfügte Bestimmungen über dem aktuellen Willen stünden. Diese Fehlvorstellungen haben mitunter großen Einfluss auf die Entscheidung, ein entsprechendes Dokument überhaupt zu erstellen. Bei der Aufklärung zu Vorsorgedokumenten im Beratungsgespräch oder mittels Informationsformaten jeglicher Art ist es deshalb wichtig, diese „Mythen“ gezielt aufzugreifen und zu berichtigen, weil sie Einfluss auf die Ausübung der Selbstbestimmung nehmen können.

Die hohen Quoten korrekter Antworten zum Fallbeispiel lassen den Schluss zu, dass die realen Konsequenzen einer Anwendung von Vorsorgedokumenten weitgehend korrekt eingeordnet werden. Bei der Fragenkonzeption wurde hierfür bewusst eingesteuert, dass die korrekte Antwort im Verlauf des Fallbeispiels teilweise mit negativen Konsequenzen für die Beispielpatientin einhergeht, um die Tragweite solcher Entscheidungen zu verdeutlichen. Ebenso wurden die Definitionen der 3 Vorsorgedokumente und in 2 von 3 Fällen die Zulässigkeit der Sterbehilfearten mehrheitlich korrekt zugeordnet. Die Fallbeschreibung zur Sterbehilfe durch Behandlungsabbruch (früher: passiv) wurde von den meisten Befragten fälschlicherweise als rechtlich unzulässig bewertet, was sich möglicherweise durch die scheinbare ärztliche Dominanz bei der Entscheidungsfindung zur Therapiezieländerung erklären lässt. Ähnliche Vorbehalte gegenüber rein ärztlich verantworteten Entscheidungen bei Wegfall der medizinischen Indikation ohne Erwähnung des Patientenwillens finden sich in der häufigen Fehlannahme, Ärzt:innen müssten bei fehlender Patientenverfügung immer lebenserhaltend behandeln, und unterstreichen das Bedürfnis der Allgemeinheit nach Selbstbestimmung auch im einwilligungsunfähigen Zustand [27].

### Limitationen und Stärken der Studie

Aufgrund der regionalen Rekrutierung und der Ungleichverteilung der Geschlechter ist die Stichprobe nicht repräsentativ, auch wenn die Altersgruppen zu den in Deutschland vorherrschenden ähnlichen Relationen vertreten sind [28]. Personen mit (Fach‑)Hochschulabschluss sind zudem mit 28 % gegenüber 18 % in der Allgemeinbevölkerung überrepräsentiert [29]. Die Ergebnisse zur hohen Prävalenz von erstellten Vorsorgedokumenten sollten aufgrund der Stichprobenabhängigkeit der Angaben in einer repräsentativen Studie überprüft werden. Es ist davon auszugehen, dass überdurchschnittlich gut informierte Teilnehmende eher den Fragebogen ausgefüllt haben, wobei die Angaben eines eher geringen Informationsstands sowie die zu anderen Studien ähnlichen Prävalenzen an Vorsorgedokumenten nicht darauf hindeuten. Sollten vorranging besonders gut Informierte die Befragung bearbeitet haben, ist von einer Überschätzung an Wissen und einer Unterschätzung des Informationsbedarfs in Bezug auf die Allgemeinbevölkerung auszugehen. Ein relevanter Non-Response-Bias lässt sich durch die hohe Abbruchquote in der Online-Variante der Befragung vermuten. Da der Fragebogen primär online abrufbar war, ist davon auszugehen, dass die Hürde zur Teilnahme für digital naive Bürger:innen höher war.

Bei der Interpretation der Fragen zu den einzelnen Dokumenttypen ist zu beachten, dass die Vorsorgevollmacht nur 84 % und die Betreuungsverfügung 61 % der Teilnehmenden geläufig waren und von diesen trotzdem alle folgenden Fragen beantwortet wurden. Zudem kann Verhalten der sozialen Erwünschtheit bei Fragen mit Selbstangaben nicht ausgeschlossen werden. Generell ist bei allen Fragen mit überdurchschnittlich hohen und niedrigen Quoten korrekter Antworten der Einfluss der Formulierung diskussionswürdig. Dagegen lässt sich anführen, dass die diesen Fragen zugrunde liegenden Fehlvorstellungen als Ergebnis der Literatur- und Internetrecherche bereits evident waren. Zur Bestätigung der Ergebnisse wären Studien mit größeren, überregionalen Stichproben sinnvoll.

Eine Stärke der Studie liegt in der breiten Rekrutierung von Bürger:innen aller Altersgruppen und Bildungsniveaus. Erstmals und insbesondere über 10 Jahre nach Inkrafttreten der gesetzlichen Regelung zur Patientenverfügung wurde mittels eines auf das deutsche Rechtssystem abgestimmten Wissenstests der tatsächliche Wissensstand zu Vorsorgedokumenten erfasst, der die Bestätigung bzw. Entkräftigung verbreiteter Fehlvorstellungen ermöglicht. Die Korrelation zwischen subjektivem und objektivem Wissensstand verdeutlicht die realistische Einschätzung des Wissens vonseiten der Befragten. Die Befunde über Wissensdefizite zu Vorsorgedokumenten bilden eine fundierte Grundlage für die Gestaltung von Informationsangeboten.

### Informationsangebote zu Vorsorgedokumenten

Während in der vorliegenden Untersuchung Defizite im Bereich des Faktenwissens auszumachen waren, beeindruckte die im Vergleich hierzu höhere Kompetenz im Anwendungswissen im Zusammenhang mit den Fallbeispielen. „Alltagstaugliches“ Wissen ist also vorhanden, weil bestimmte Situationen schon erlebt bzw. durchdacht wurden. Hieraus lässt sich ableiten, dass sich Informationsangebote für die breite Allgemeinbevölkerung nicht auf die reine Informationsbereitstellung, wie es sie bereits in vielfältiger Form gibt, beschränken sollten. Vielmehr müssen sie einen Bezug zum Alltag der Menschen aufweisen und diese zudem emotional erreichen, z. B. damit das Thema „gesundheitliche Vorausplanung“ nicht weiter vor sich hergeschoben wird.

Das Vorhandensein gewisser Alltagskompetenzen und Kenntnisse sollte als Ausgangsbasis für Initiativen zur Steigerung des Wissens dienen. (Erfahrungs‑)Wissen in Bezug auf Anwendungsszenarien mag manche Menschen in einer falschen Sicherheit wiegen, wenn es nicht auf die eigenen Optionen der gesundheitlichen Selbstbestimmung übertragen wird. Formen der Wissensvermittlung, die über die reine Bereitstellung von Informationen hinausgehen, sollten den Einsatz von Fallszenarien, Videos und interaktiven Elementen mit Bezug zum vorhandenen Alltags- und Erfahrungswissen beinhalten, um möglichst niederschwellig die Scheu zu reduzieren, sich mit diesem ernsten Thema eingehend zu beschäftigen. Die Zielgruppe sollten Bürger:innen jeden Alters und sozialer Herkunft bilden, unabhängig von individuellen Krankheitserfahrungen oder Vorerfahrungen mit Vorsorgedokumenten.

Vor dem Hintergrund, dass Vorsorgedokumente in der Praxis eine wesentliche Orientierung für Behandlungsentscheidungen bei äußerst vulnerablen Personen darstellen, sollten hohe Standards an die Beratung und an jegliche Informationsmedien zur Erstellung von Vorsorgedokumenten angelegt werden. Menschen mit Beratungsfunktion zu Vorsorgedokumenten, aus dem ärztlichen wie auch aus dem nicht-ärztlichen Bereich, können die verbreiteten Fehlvorstellungen dabei als Anhaltspunkt für die Beratungsinhalte dienen.

Dem Anspruch einer umfassenden, an den Bedürfnissen der Betroffenen orientierten Beratung versucht das Konzept „Advance Care Planning“ (ACP) nachzukommen. ACP versteht sich unter Bezugnahme auf die Konsensus-Definition von Rietjens et al. (2017) als ein dynamischer Gesprächsprozess, der Betroffene dazu befähigt, ihre Ziele und Präferenzen in Bezug auf ihre zukünftige Versorgung bzw. im Hinblick auf eine bestimmte Behandlung zu reflektieren und zu dokumentierten [30, 31]. Die Umsetzung von ACP erfolgt national wie international mit unterschiedlichen Ansätzen. Umfassende Programme beispielsweise in Australien oder teilweise in Kanada richten sich an die breite Bevölkerung und inkludieren alle Altersgruppen und Menschen in diversen Lebenslagen [32]. In Deutschland wird über § 132g SGB V (Soziales Gesetzbuch) ein besonderer finanzieller Anreiz gesetzt, ACP in Pflegeeinrichtungen und Einrichtungen der Eingliederungshilfe für behinderte Menschen mittels Gesprächsbegleiter anzubieten [33]. Will man die gesundheitliche Vorausplanung der Bürger:innen jedoch im Allgemeinen stärken und nicht auf bestimmte Personengruppen oder Settings beschränken, bedarf es groß angelegter ACP-Programme, wie sie eben in Australien vorhanden sind. Schulungsmaterialien, Videos, Lernforen und Erfahrungsberichte sollten auch die Menschen adressieren, die aus völliger Gesundheit heraus Vorsorge treffen wollen oder beispielsweise als bevollmächtigte Personen dem Willen einer nahestehenden betroffenen Person Ausdruck verleihen müssen.

Greift man auf die Begriffsbeschreibung von „Gesundheitskompetenz“ zurück, umfasst diese das Wissen, die Motivation und die Fähigkeiten von Menschen, relevante Gesundheitsinformationen zu finden, zu verstehen, zu beurteilen und im Alltag anzuwenden [34]. Um all diesen Dimensionen der Gesundheitskompetenz begegnen zu können, bedarf es leicht zugänglicher, zielgruppenorientierter Lernformate mit unterschiedlichem Detaillierungsgrad, in etwa durch das Platzieren der Themen in der Öffentlichkeit oder das Zugänglichmachen von Informationsangeboten auf Portalen wie dem Gesundheitsportal. Ob dieses im Rahmen von ACP oder über andere (parallele) Ansätze geschieht, mag dahingestellt sein. Das Ziel muss sein, in der hier angesprochenen großen Gruppe von Bürger:innen einen maximalen Wissenszuwachs zu erreichen. Letztlich gilt, dass Selbstbestimmung in der Behandlung oder gar am Lebensende nur gelingen kann, wenn ausreichendes Wissen und Verständnis vorhanden sind.

## Fazit

Die Studie erfasst erstmals den objektiven und subjektiven Wissensstand von Bürger:innen zu Vorsorgedokumenten. Fragen zur Anwendung der Dokumente anhand eines Fallbeispiels werden weitaus häufiger korrekt beantwortet als Fragen zum faktischen Wissen über die Dokumenttypen selbst. Aus den Ergebnissen lässt sich ableiten, dass die große Tragweite der auf Vorsorgedokumenten basierenden Behandlungsentscheidungen verstanden wird, bis hin zu sehr konkreten Maßnahmen, die zum Tod des betroffenen Menschen führen können. Zudem wird die Bedeutung der Vorsorgedokumente mehrheitlich als sehr hoch angesehen und eine bessere Informiertheit angestrebt. Gleichzeitig wird der eigene Wissensstand als niedrig eingeschätzt und es werden Unsicherheiten von der Erstellung der Dokumente bis hin zu deren Anwendung bekundet. Das Feld möglicher Informationsquellen oder beratender Personen/Institutionen ist weit und spiegelt unterschiedliche Interessen wider.

Da potenziell jeder Erwachsene eine Vorausplanung über Vorsorgedokumente treffen kann und sich in vielen Fällen im Laufe seines Lebens irgendwann durch Geschehnisse im Familien- oder Bekanntenkreis mit dem Thema konfrontiert sieht, sind die Ergebnisse dieser Studie von hoher Relevanz und zeigen zugleich die Notwendigkeit von strukturierten und niederschwelligen Informationsangeboten auf. Damit sollte der Offenheit aller im Feld involvierten Akteure, sich dem Thema und dem Wunsch nach Informationen anzunehmen, begegnet werden.

### Supplementary Information




