# Supplementary material for: Knowledge and misconceptions on advance directives—results of a citizen survey
Source: Bundesgesundheitsblatt Gesundheitsforschung Gesundheitsschutz. 2023 Aug 17;66(10):1172–82. [Article in German] doi: 10.1007/s00103-023-03751-y (PMC10539417; doi:10.1007/s00103-023-03751-y)
Supplement: Supplementary file 1 [file 103_2023_3751_MOESM1_ESM.pdf]

Ausschnitt aus dem Original-Fragebogen

Teil 4: Wissen zu Vorsorgedokumenten

Kreuzen Sie jeweils an, welche Antwort Sie für richtig halten. Wenn Sie die Antwort nicht wissen, wählen Sie bitte „Weiß nicht“.

4.1. Definitionen der Vorsorgedokumente

28	Hiermit können Sie im Voraus festlegen, ob bestimmte medizinische Maßnahmen durchzuführen oder zu unterlassen sind, falls Sie aufgrund eines Unfalls oder einer Erkrankungen nicht mehr selbst entscheiden können.	O Patientenverfügung<br>O Vorsorgevollmacht<br>O Betreuungsverfügung<br>O Weiß nicht
29	Hiermit können Sie bestimmen, wer im Falle Ihrer Einwilligungs- oder Geschäftsunfähigkeit vom Gericht als Betreuer/in bestellt werden soll, sofern keine weiteren Vorkehrungen getroffen wurden.	O Patientenverfügung<br>O Vorsorgevollmacht<br>O Betreuungsverfügung<br>O Weiß nicht
30	Hiermit bestimmen Sie eine Person, die stellvertretend für Sie Entscheidungen treffen kann, wenn Sie dazu nicht mehr in der Lage sind.	O Patientenverfügung<br>O Vorsorgevollmacht<br>O Betreuungsverfügung<br>O Weiß nicht

4.2. Spezifischere Fragen zu Vorsorgedokumenten

31	Vorsorgedokumente dienen dazu, den Willen eines Menschen für zukünftige Entscheidungen, die der Mensch nicht mehr selbst treffen kann, festzuhalten.	O Richtig<br>O Falsch<br>O Weiß nicht
32	Jede*r Bürger*in kann ab Volljährigkeit ein gültiges Vorsorgedokument erstellen.	O Richtig<br>O Falsch<br>O Weiß nicht
33	Ein Mensch mit demenzieller Erkrankung kann grundsätzlich ab der Diagnose kein gültiges Vorsorgedokument mehr erstellen.	O Richtig<br>O Falsch<br>O Weiß nicht
34	Die ärztliche Schweigepflicht gilt nicht gegenüber Ehegatten.	O Richtig<br>O Falsch<br>O Weiß nicht

Information: Die Patientenverfügung

- gilt für den Fall späterer Einwilligungsunfähigkeit
- bestimmt im Voraus die Einwilligung oder Nichteinwilligung in medizinische Behandlungsmaßnahmen
- legt die Lebens- bzw. Krankheitssituationen (z.B. aufgrund von Unfall oder Krankheit) fest, in denen die Verfügung gelten soll

(Bitte korrigieren Sie Ihre vorher gegebenen Antworten nicht!)

35	Wenn keine Patientenverfügung vorliegt, müssen Ärzt*innen immer lebenserhaltend behandeln.	O Richtig<br>O Falsch<br>O Weiß nicht

36	Die Patientenverfügung muss durch eine/n Zeugen/Zeugin durch Unterschrift bestätigt werden.	0 Richtig<br>0 Falsch<br>0 Weiß nicht
37	Eine notarielle Beglaubigung der Verfügung ist nicht notwendig.	0 Richtig<br>0 Falsch<br>0 Weiß nicht
38	Ab dem Zeitpunkt der Erstellung steht der in der Patientenverfügung verschriftlichte Wille über dem aktuell geäußerten Willen.	0 Richtig<br>0 Falsch<br>0 Weiß nicht
39	Bisher fehlt es an einer gesetzlichen Grundlage zum Umgang mit Patientenverfügungen in Deutschland.	0 Richtig<br>0 Falsch<br>0 Weiß nicht
40	Eine Patientenverfügung wird ausschließlich dann wirksam, wenn sich der/die Betroffene im Endstadium einer tödlichen Erkrankung befindet.	0 Richtig<br>0 Falsch<br>0 Weiß nicht
41	Eine Patientenverfügung ist auch ohne regelmäßige Aktualisierung gültig, selbst nach 20 Jahren.	0 Richtig<br>0 Falsch<br>0 Weiß nicht
42	Mit einer Patientenverfügung kann man nur medizinische Maßnahmen ablehnen.	0 Richtig<br>0 Falsch<br>0 Weiß nicht

Information: Die Vorsorgevollmacht

- wird (in der Regel erst) wirksam bei Einwilligungsunfähigkeit
- bestimmt im Voraus eine oder mehrere Personen, die stellvertretend für Sie Entscheidungen in festgelegten Lebensbereichen treffen kann/können, bspw. in Gesundheits- und Vermögensangelegenheiten

43	Wenn man eine Vorsorgevollmacht hat, muss kein/e gesetzliche/r Betreuer*in mehr bestellt werden.	0 Richtig<br>0 Falsch<br>0 Weiß nicht
44	Der/die Bevollmächtigte ist verpflichtet nach dem Willen des/der Patienten/Patientin zu handeln.	0 Richtig<br>0 Falsch<br>0 Weiß nicht
45	Der/die Bevollmächtigte in Gesundheitsangelegenheiten darf darüber entscheiden, ob lebenserhaltende Maßnahmen eingestellt werden.	0 Richtig<br>0 Falsch<br>0 Weiß nicht
46	Mit einer Vorsorgevollmacht kann bestimmt werden, ob die bevollmächtigte Person auch über freiheitsentziehende Maßnahmen entscheiden darf (z.B. zwangsweise Unterbringung in Psychiatrie, Fixierung/Festbinden).	0 Richtig<br>0 Falsch<br>0 Weiß nicht

Information: Die Betreuungsverfügung

- Gilt für den späteren Fall einer Einwilligungsunfähigkeit
- bestimmt im Voraus eine oder mehrere Personen, die vom Betreuungsgericht als rechtliche Betreuer bestellt werden sollen

47	Die gerichtliche Bestellung eines rechtlichen Betreuers kommt einer Entmündigung gleich.	0 Richtig<br>0 Falsch<br>0 Weiß nicht

48	Jede/r kann seine/n Betreuer/in frei festlegen.	0 Richtig<br>0 Falsch<br>0 Weiß nicht
49	Eine Entscheidung des Betreuungsgerichts ist nur nötig, wenn sich Betreuer/in und behandelnde Ärzt*in nicht einig sind, ob eine Maßnahme dem Willen des Patienten entspricht.	0 Richtig<br>0 Falsch<br>0 Weiß nicht

4.3 Fallbeispiel: Es folgt ein Fall zur Anwendung von Vorsorgedokumenten. Bitte entscheiden Sie für jede Situation, ob Sie die Aussagen für richtig oder falsch halten. Wenn Sie sich unsicher sind, wählen Sie bitte „Weiß nicht“.

Eine 81-jährige Frau erleidet mehrere Schlaganfälle. Als Folge ist sie blind, linksseitig gelähmt, nicht in der Lage zu sitzen und geistig nicht orientiert. Ein Gespräch ist nicht möglich. Sie kann keine Nahrung schlucken. Ohne ausreichende Nahrungs- und Flüssigkeitszufuhr wird sie binnen circa sechs Wochen versterben. Der behandelnde Arzt im Krankenhaus schlägt vor, mittels einer Operation eine Magensonde über die Bauchdecke einzuführen, um die Versorgung sicherzustellen.<br>Der Sohn wendet jedoch ein, dass seine Mutter einen solchen Zustand niemals gewollt hätte.		
	Wie hätte die Frau schon <u>vor</u> der plötzlichen Erkrankung Vorsorge treffen können?	
50	Gar nicht. Die Ärzt*innen müssen eine Magensode legen, da sonst Lebensgefahr besteht.	0 Richtig<br>0 Falsch<br>0 Weiß nicht
51	Mithilfe einer Patientenverfügung hätte sie das Legen der Magensonde ablehnen können.	0 Richtig<br>0 Falsch<br>0 Weiß nicht
52	Mithilfe einer Vorsorgevollmacht hätte sie eine/n Bevollmächtigte/n bestimmen können, der/die ihre Wünsche kennt und in ihrem Namen den Eingriff ablehnt.	0 Richtig<br>0 Falsch<br>0 Weiß nicht
	Angenommen, die Frau hat eine Patientenverfügung erstellt. Darin steht, dass sie im Falle einer schweren Pflegebedürftigkeit keine künstliche Ernährung (Magensonde) wünscht.<br>Was meinen Sie: Wie müssen die Ärzt*innen nun damit verfahren?	
53	Bei eindeutigen Aussagen in der Patientenverfügung sind die Ärzt*innen zur Umsetzung der beschriebenen Willensäußerungen verpflichtet.	0 Richtig<br>0 Falsch<br>0 Weiß nicht
54	Die Frau hatte die Patientenverfügung <u>vor</u> ihrer schweren plötzlichen Krankheit erstellt. Deshalb wird sie in dieser Situation nicht wirksam.	0 Richtig<br>0 Falsch<br>0 Weiß nicht
55	Die niedergeschriebenen Willensäußerungen sind wirksam, wenn wie hier die aktuelle Lebenssituation der in der Patientenverfügung beschriebenen Lebenssituation entspricht.	0 Richtig<br>0 Falsch<br>0 Weiß nicht
56	Die medizinische Notwendigkeit der Behandlung steht an erster Stelle. Die Willenserklärung ist deshalb nachrangig.	0 Richtig<br>0 Falsch<br>0 Weiß nicht
	Welche Möglichkeiten bestehen in diesem Fall, wenn - dem Wunsch der Frau folgend - keine Magensonde gelegt werden würde?	
57	Die Ernährung wird eingestellt. Es werden pflegerische Maßnahmen durchgeführt und Leiden (Hunger/Durst) soweit wie möglich gelindert bis der Tod eintritt.	0 Richtig<br>0 Falsch<br>0 Weiß nicht

58	Um das Sterben nicht unnötig in die Länge zu ziehen, können die Ärzt*innen ein starkes Schlafmittel spritzen, welches den Todeseintritt angenehmer macht und schneller herbeiführt.	0 Richtig<br>0 Falsch<br>0 Weiß nicht

4.4. Zulässigkeit der Sterbehilfe: Nachfolgend werden 3 Beispiele von Sterbehilfe beschrieben. Die Einwilligung der Patient*innen ist jeweils Voraussetzung für die Maßnahmen. Kreuzen Sie bitte jeweils an, ob Sie das Vorgehen nach deutscher Gesetzeslage für rechtlich zulässig halten oder für verboten.

59	Die Atemnot eines 75-jährigen Covid-19-Patienten verschlechtert sich so stark, dass er künstlich beatmet werden muss. Im weiteren Verlauf zeigt sich aber, dass ein Überleben nicht realistisch ist. Um das Leiden nicht in die Länge zu ziehen, beschließen die Ärzt*innen die Beatmungsmaschine abzuschalten. Der Patient verstirbt daraufhin.	0 Zulässig<br>0 Verboten<br>0 Weiß nicht
60	Auf der Palliativstation liegt eine 56-jährige Patientin mit Brustkrebs im Endstadium. Die Absiedelungen der Krebszellen in den Knochen bereiten ihr starke Schmerzen. Als absehbar wird, dass der Tod innerhalb der nächsten Tage eintreten wird und die Patientin vor Schmerzen stöhnt und keine Ruhe findet, verabreicht der Stationsarzt ein starkes Schmerzmittel (Morphium) zur Schmerzlinderung. Er weiß und nimmt hierbei in Kauf, dass die Patientin durch die hohe Dosis früher versterben könnte als es der natürliche Krankheitsverlauf vorsieht, beabsichtigt dieses jedoch nicht.	0 Zulässig<br>0 Verboten<br>0 Weiß nicht
61	Nach einem Verkehrsunfall mit schwerer Kopfverletzung erwacht ein 36-jähriger Mann mit schweren Lähmungen aller Gliedmaßen. Auch nach mehreren Monaten in einer Rehaklinik ist keine Besserung in Aussicht. Da er sich ein solches Leben auf Dauer nicht vorstellen kann, bittet er wiederholt und nachdrücklich die Ärzt*innen ihm zu einem friedvollen Sterben zu verhelfen. Die Ärzt*innen kommen seinem Willen nach und spritzen ihm ein hochdosiertes Schlafmittel, welches den Tod durch Atemstillstand herbeiführt.	0 Zulässig<br>0 Verboten<br>0 Weiß nicht

In der Zusammenschau: Wie leicht bzw. schwer fiel es Ihnen die Fragen aus dem Wissenstest in Teil 4 zu beantworten?				
62	☐<br>schwer	☐<br>eher schwer	☐<br>eher leicht	☐<br>leicht
63	Wie viel Prozent der 34 Fragen glauben Sie richtig beantwortet zu haben („Weiß nicht" zählt als falsche Antwort)? ________ %			
64	Von diesen richtig beantworteten Fragen: Wie viele glauben Sie durch **Raten** und wie viele durch **Vorwissen** richtig beantwortet zu haben (insg. 100%)?<br><br>________ % durch Raten<br><br>________ % durch Vorwissen			
